# Supplementary material for: Reviewing the Effects of Skin Manipulations on Adult Newt Limb Regeneration: Implications for the Subcutaneous Origin of Axial Pattern Formation
Source: Biomedicines. 2021 Oct 9;9(10):1426. doi: 10.3390/biomedicines9101426 (PMC8533417; doi:10.3390/biomedicines9101426)
Supplement: Supplementary file 1 [file biomedicines-09-01426-s001.zip › biomedicines-1390138-supplementary.pdf]

Supplementary Information

for

Reviewing the Effects of Skin Manipulations on Adult Newt Limb Regeneration: Implications for the Subcutaneous Origin of Axial Pattern Formation

Martin Miguel Casco-Robles, Kayo Yasuda, Kensuke Yahata, Fumiaki Maruo & Chikafumi Chiba
CC: chichiba@biol.tsukuba.ac.jp

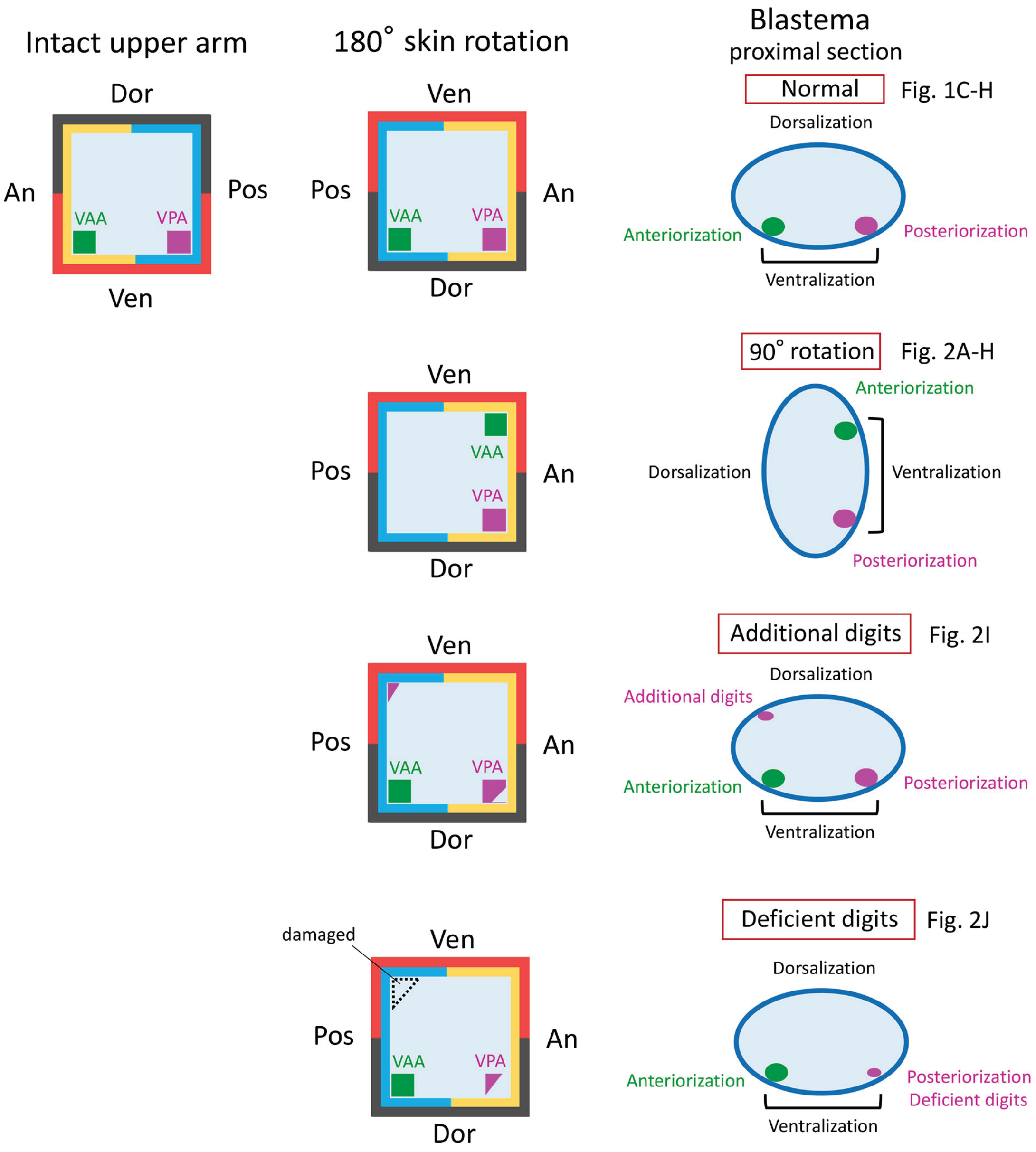

Figure S1. Hypothetical mechanisms of abnormalities in 180° skin rotation.

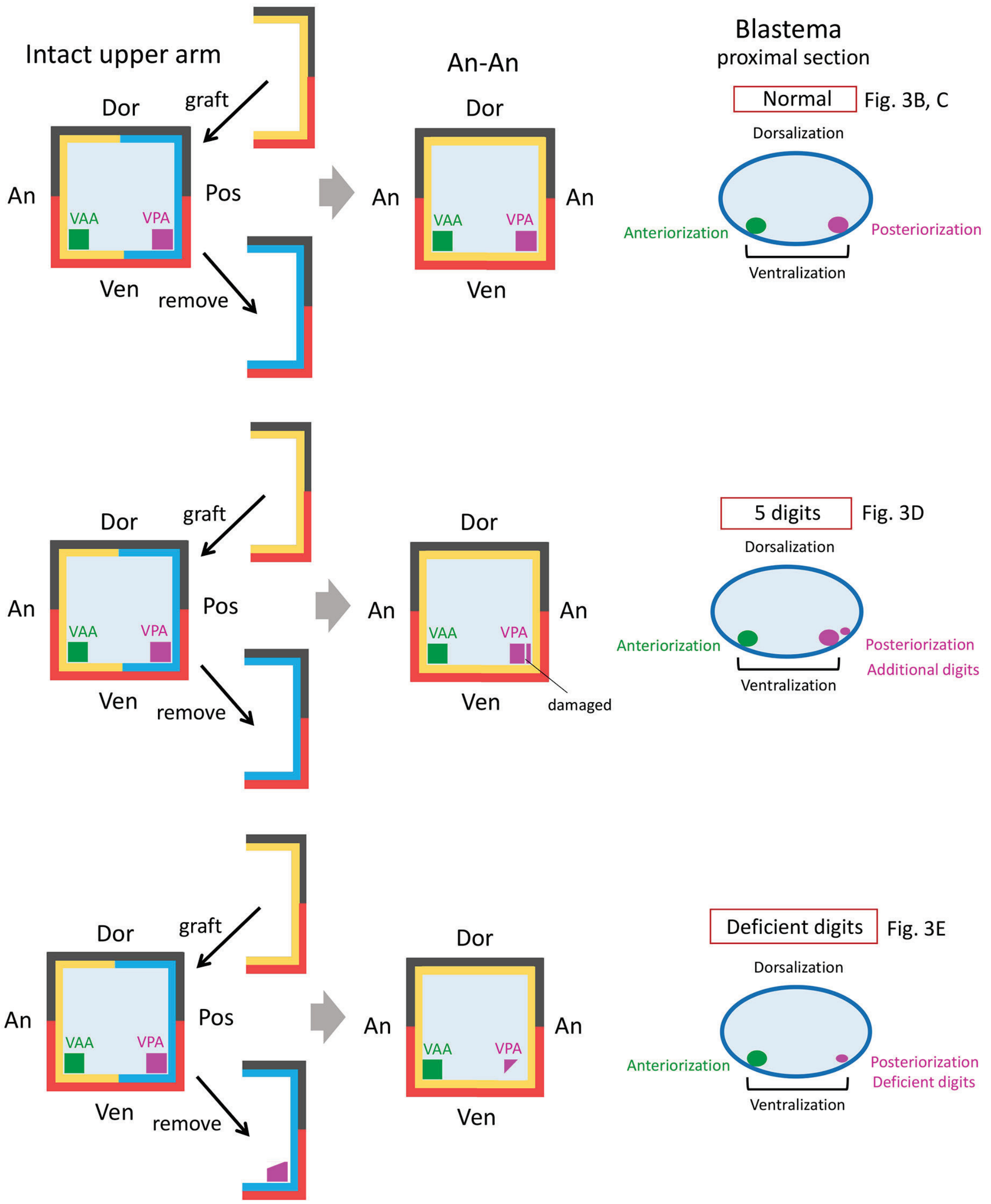

Figure S2. Hypothetical mechanisms of abnormalities in the An-An pattern of half-skin graft operation.

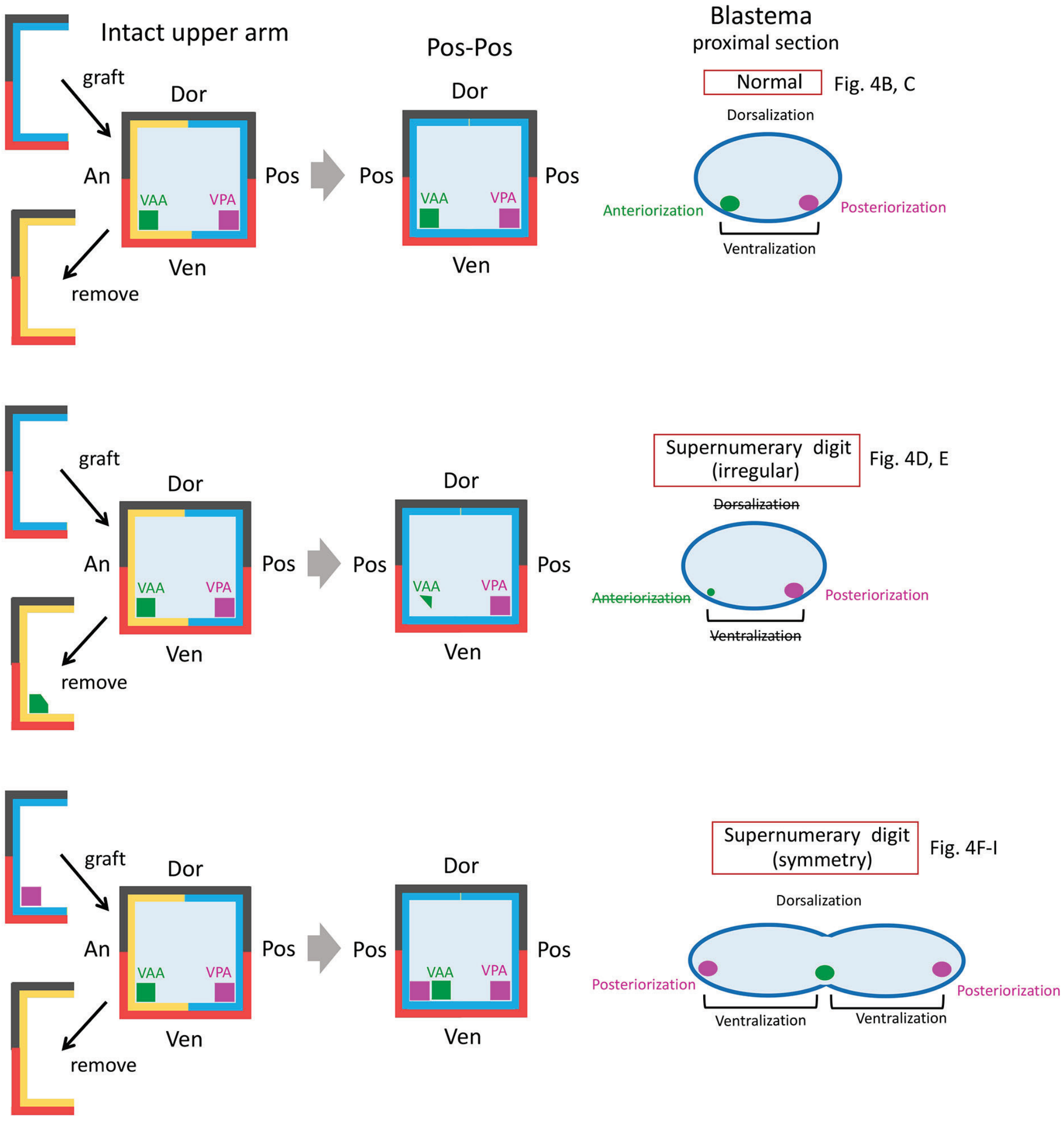

Figure S3. Hypothetical mechanisms of abnormalities in the Pos-Pos pattern of half-skin graft operation.

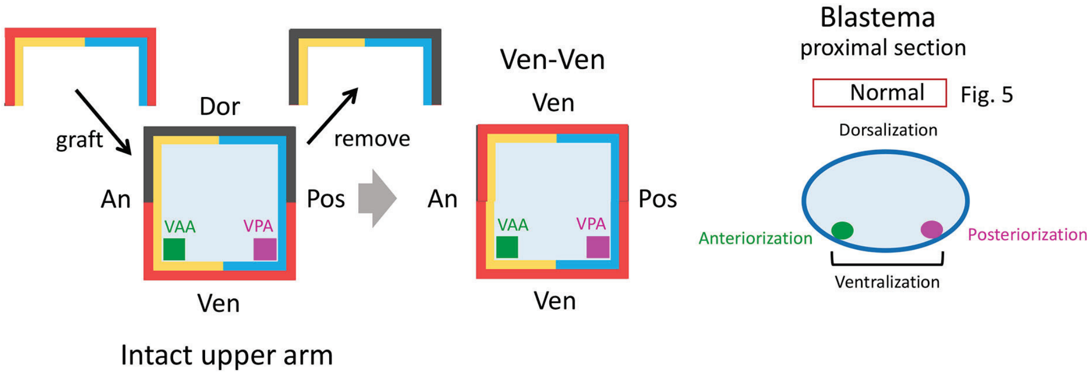

Figure S4. Hypothetical mechanisms of abnormalities in the Ven-Ven pattern of half-skin graft operation.

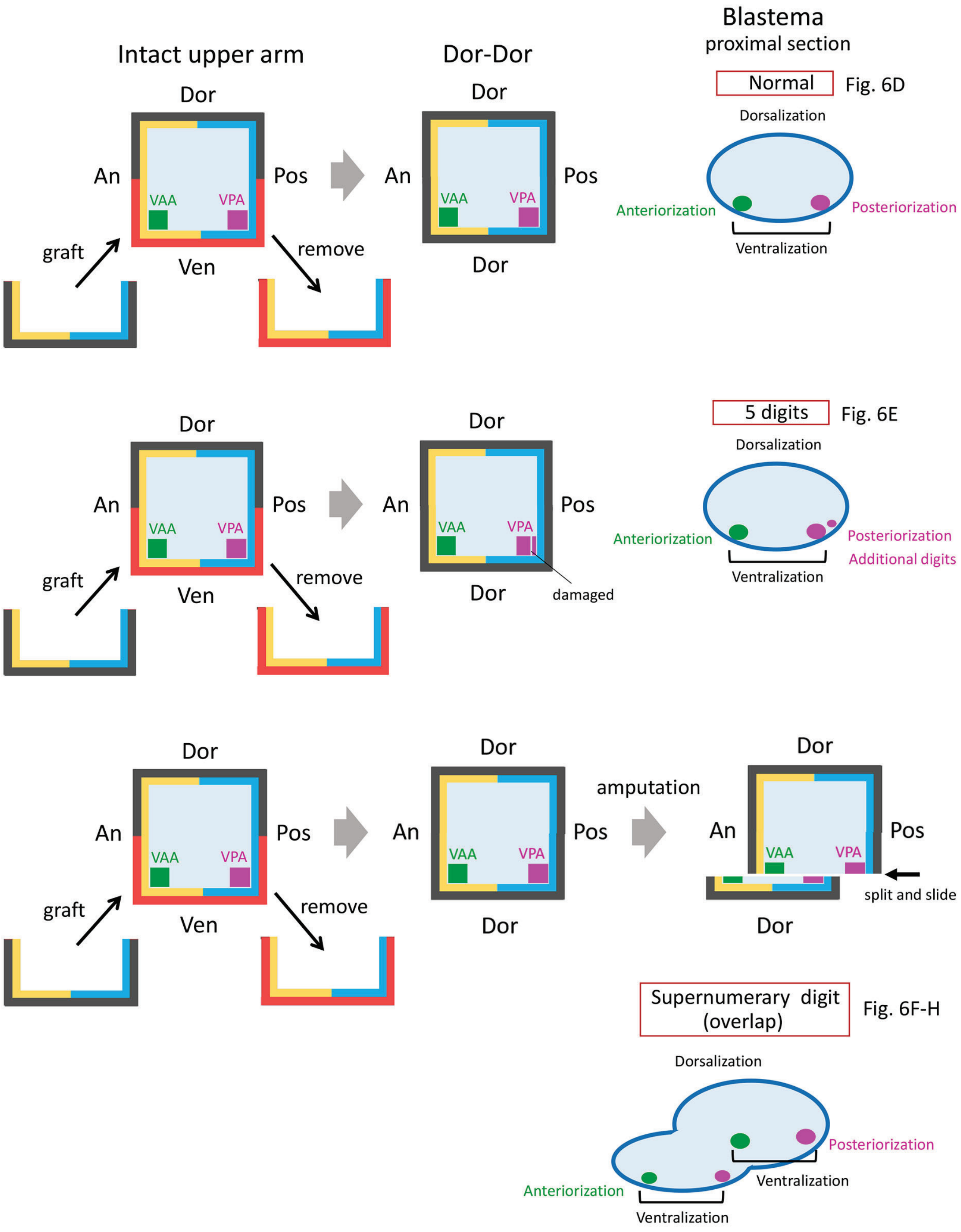

Figure S5. Hypothetical mechanisms of abnormalities in the Dor-Dor pattern of half-skin graft operation.

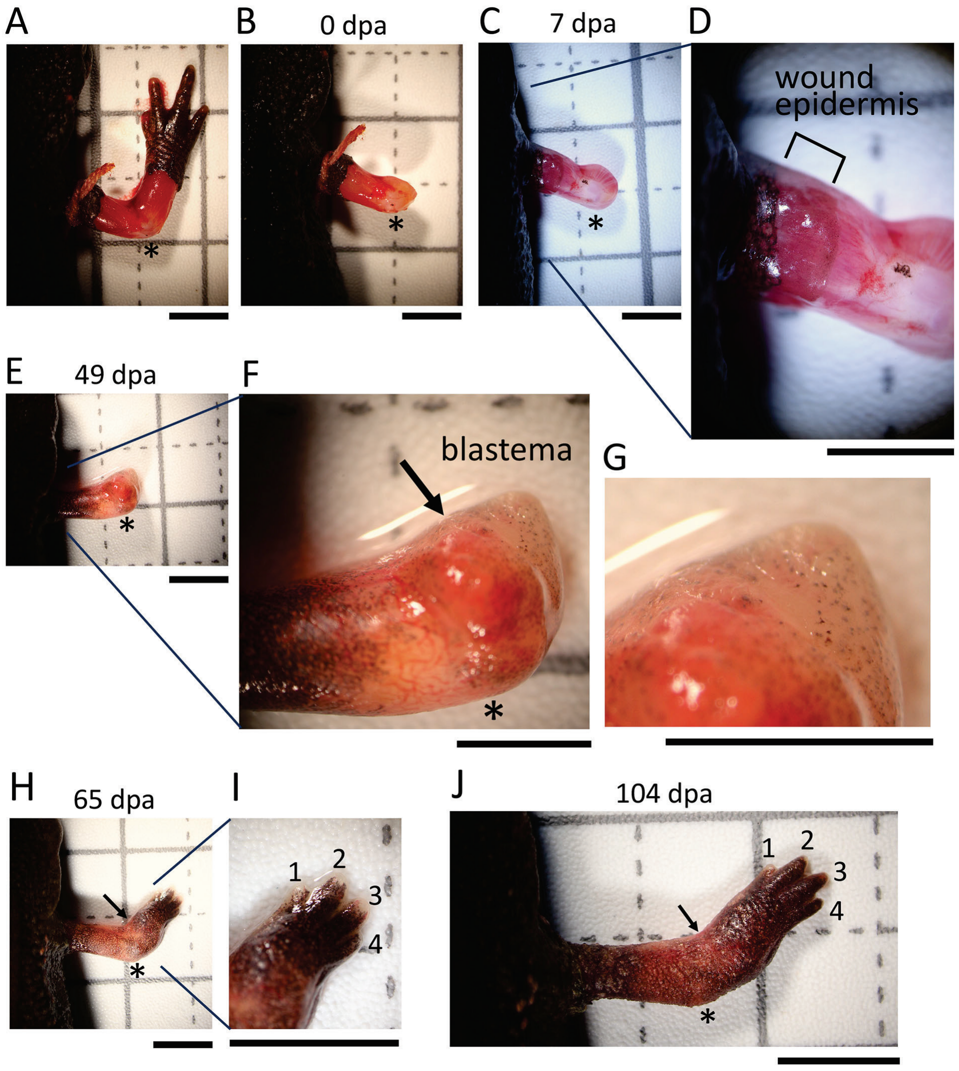

Figure S6. Limb regeneration in the skin-less model. (**A**) A forelimb immediately after removal of skin. To control bleeding during the operation, a small piece of string was tightened around the base of the limb as a tourniquet. (**B**) The limb in **A** immediately after amputation (0 dpa) below the elbow. The tourniquet was kept for 24 h and animals were reared at 18 C°. (**C**, **D**). 7 dpa. Wound epidermis grew from the wound edge of skin. (**E**-**G**) 49 dpa. The blastema grew to a stage just before the cartilage began to differentiate with a pattern. Note that a large number of pigment cells were present under the wound epidermis surrounding the blastema. (**H**, **I**) 65 dpa. (**J**) 104 dpa. The number near the digit indicates digit number. The asterisk indicates the elbow. The arrow indicates the amputation position. dpa: day post amputation. Scale bars: 5 mm (**A-C**, **E**, **H-J**); 2.5 mm (**D**, **F**, **G**). In this model, a normally patterned limb was regenerated (n=3), although blastema formation did not begin until the exposed tissue was completely covered by the epidermis extending from the wound edge of the skin on the upper arm. We first anticipated that by the time the dermal mesenchymal cells in the skin of the upper arm arrived at the amputation site on the lower arm, blastema formation would have already begun. However, by the time the blastema began to form, pigment cells which were originally located at the epidermis-dermis boundary of the skin had migrated from the wound edge of the skin on the upper arm to the blastema. Presumably, the earlier mesenchymal cells of the dermis had also arrived by that time. Therefore, in this model, we cannot rule out the possibility that dermis-originating mesenchymal cells might be involved in pattern formation of the blastema. However, if this is the case, dermal mesenchymal cells might have to adjust their geometrical identity to the destination as they migrate.

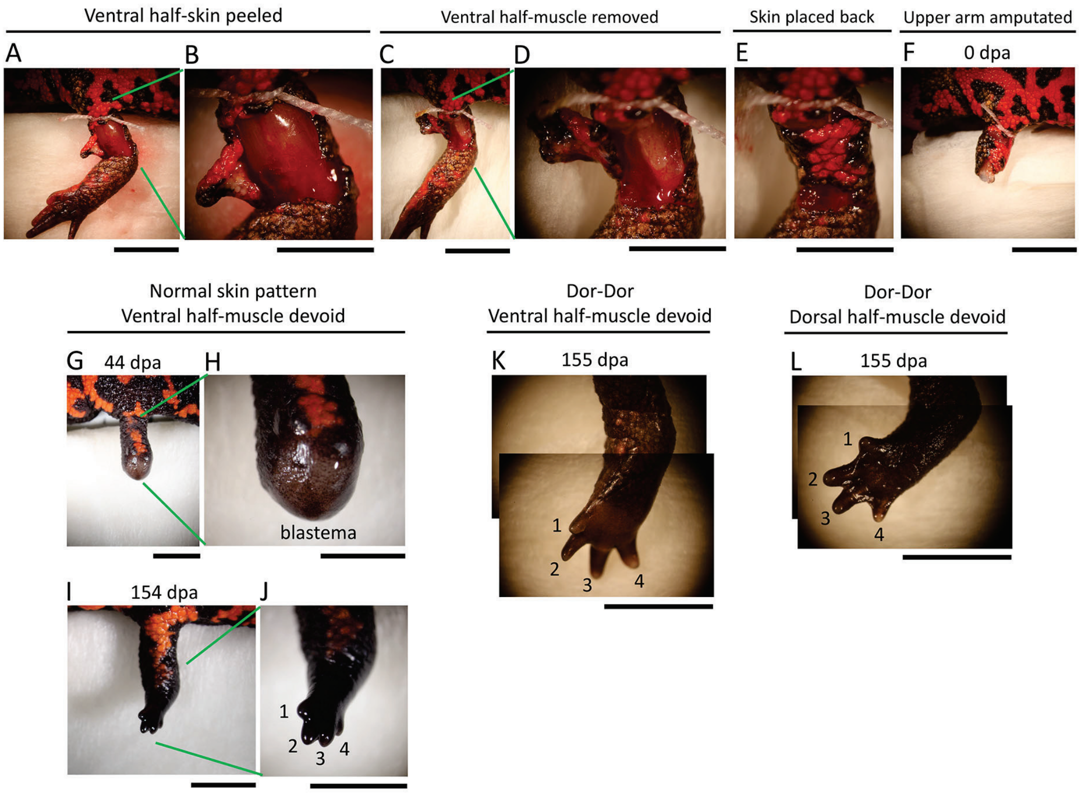

Figure S7. Limb regeneration in the subcutaneous tissue-less model. (**A**-**F**) Operation. (**A**, **B**) The ventral half-skin of the upper arm was peeled. The tissue under the skin, namely the subcutaneous tissue, was exposed. Note that in the adult newt forelimbs, subcutaneous tissue was a thin connective tissue layer that surrounded the muscles, in which nerves and blood/lymphatic capillaries were embedded. To control bleeding during the operation, a small piece of string was tightened around the base of the limb as a tourniquet. (**C**, **D**) The ventral half-muscle was excised together with surrounding subcutaneous tissue. (**E**) The skin flap was placed back. (**F**) The upper arm was amputated along the distal margin of the skin flap (0 dpa). The tourniquet was kept for 24 h and animals were reared at 18 C°. (**G**-**J**) Representative case showing regeneration of a ventral half-muscle devoid limb with a normal skin pattern. Operation was made as in **A**-**F**. (**G**, **H**) 44 dpa. Blastema was formed. At this stage, a large number of pigment cells originating from the skin gathered under the epidermis surrounding the blastema (n=3). (**I**, **J**) 154 dpa. A normally patterned limb had regenerated (n=3). Note that the animals in **G** and **I** were different. (**K**) Representative case showing regeneration of a ventral half-muscle devoid limb with Dor-Dor skin pattern at 155 dpa. Half-skin graft operation in Dor-Dor pattern was combined with the ventral half-subcutaneous tissue removal operation. A normally patterned limb was regenerated (n=3). (**L**) Representative case showing regeneration of a dorsal half-muscle devoid limb with the Dor-Dor skin pattern at 155 dpa. Half-skin graft operation in the Dor-Dor pattern was combined with the dorsal half-subcutaneous tissue removal operation. A normally patterned limb had regenerated (n=3). The number near the digit indicates digit number. dpa: day post amputation. Scale bars: 5 mm (**A**, **C**, **F**, **G**, **I**); 2.5 mm (**B**, **D**, **E**, **H**, **J**, **K**, **L**). In this model, surprisingly, limb regeneration proceeded with normal speed regardless of either the skin pattern (normal or Dor-Dor) or the part of the forearm (ventral half or dorsal half) from which subcutaneous tissue was removed. In all cases, as shown in **H**, pigment cells migrating from the skin started to form a layer under the epidermis surrounding the blastema, as the blastema formed. Therefore, it was difficult to rule out the possibility that mesenchymal cells migrating from the dermis in the skin had also arrived at the blastema.
